# Active Circulation of Corynebacterium ulcerans among Nonhuman Primates

**DOI:** 10.1128/spectrum.00894-22

**Published:** 2022-07-12

**Authors:** Archana Thomas, Ariel M. Slifka, Sara M. Hendrickson, Ian J. Amanna, Mark K. Slifka

**Affiliations:** a Oregon Health & Science University, Beaverton, Oregon, USA; b Najit Technologies, Inc., Beaverton, Oregon, USA; Institut National de Santé Publique du Québec

**Keywords:** *Corynebacterium*, diphtheria, nonhuman primates, neutralizing antibodies, ELISA

## Abstract

Diphtheria is rare in the United States. and many industrialized nations due to development of an effective vaccine, coupled with high vaccination coverage. Although there is continued risk of importation and transmission of Corynebacterium diphtheriae, C. ulcerans has now become the dominant source of diphtheria cases among several European countries. Bearing this in mind, a better understanding of C. ulcerans biology is clearly needed. Here, we identified active transmission of toxigenic C. ulcerans among indoor- and outdoor-housed rhesus macaques based on diphtheria toxin-specific serology assays as well as direct isolation of C. ulcerans from a recently infected animal. In addition to animal-to-animal transmission, we found serological evidence indicative of potential human transmission. Together, these results provide new details on natural Corynebacterium transmission among nonhuman primates and emphasizes the importance of maintaining high vaccination coverage to reduce the risk of potential zoonotic infection.

**IMPORTANCE** C. ulcerans represents an emerging zoonotic agent of diphtheria, but little is known about its transmission or maintenance among animal reservoirs. In these studies, we identified diphtheria outbreaks among both outdoor- and indoor-housed rhesus macaques and isolated a toxigenic strain of C. ulcerans from a recently infected animal. Retrospective analysis indicated that toxigenic *Corynebacteria* have been circulating among these primates for decades with the potential for rare zoonotic transmission to humans.

## INTRODUCTION

Corynebacterium diphtheriae is the most recognized cause of diphtheria outbreaks but other Corynebacterium, such as C. ulcerans, may also harbor diphtheria toxin genes and cause respiratory illness with similar case-fatality rates of 13–15% (1, 2). Remarkably, toxigenic C. ulcerans has become a more common cause of respiratory disease than toxigenic C. diphtheriae in Europe (1–3). Since 2009, toxigenic C. ulcerans has been reported in Germany at higher rates than toxigenic C. diphtheriae and in France, 12 of the 19 reported cases of diphtheria (63%) identified between 2002 and 2008 were attributed to C. ulcerans (3). The U.K. reported similar rates of toxigenic C. ulcerans and C. diphtheriae from 1986 to 1997 (4) yet from 1998 to 2008 (4) and from 2009 to 2017 (2) there were approximately twice as many cases of respiratory diphtheria due to toxigenic C. ulcerans compared to C. diphtheriae.

The main risk factor for C. diphtheriae infection is recent travel to an endemic country whereas C. ulcerans infections were initially associated with infected cattle (3). However, toxigenic C. ulcerans has also been isolated from a wide range of animals including cows, horses, pigs, goats, dogs, cats, camels, otters, ground squirrels, and hedgehogs (1, 3, 5). Moreover, C. ulcerans has been isolated from multiple species of nonhuman primates (NHP) including cynomolgus macaques, bonnet macaques, rhesus macaques and gray langurs (1, 6–8). C. ulcerans has been isolated from NHP immediately after capture (8) as well as at various primate centers (6–10). C. ulcerans causes asymptomatic carriage as well as clinical respiratory disease and cutaneous infection in primates (6–8) whereas C. diphtheriae and C. pseudotuberculosis infections have not been identified among NHP (6).

Among countries with high vaccination coverage, diphtheria has become exceedingly rare (11) and C. ulcerans appears to be displacing C. diphtheriae as the dominant cause of this respiratory human disease (1–4). More studies are needed to understand potential risk factors and transmission rates of Corynebacterium spp., especially C. ulcerans. Here, we identified active transmission of C. ulcerans among both indoor- and outdoor-housed rhesus macaques and it appears that toxigenic Corynebacteria may have been circulating at the Oregon National Primate Research Center (ONPRC) for several decades. Analysis of longitudinal human serum samples resulted in the serological identification of up to three potential cases of undiagnosed diphtheria among individuals who had contact with NHP or NHP samples. Together, this provides an opportunity to study the natural transmission of a human pathogen among NHP and underscores the need to maintain high vaccination coverage, especially among personnel with close contact to NHP or those who work with NHP samples.

## RESULTS

**FIG 1:**
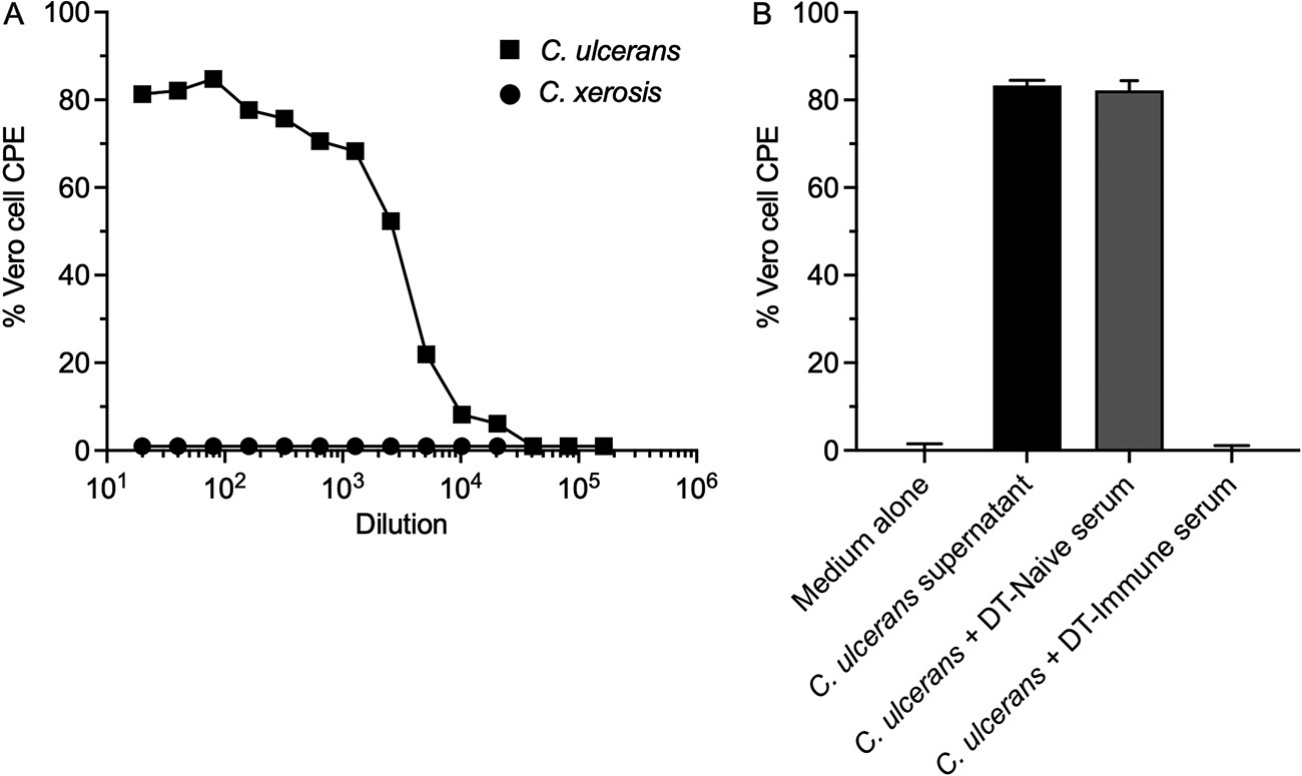
Determination of toxigenic status of C. ulcerans and *C. xerosis.* Vero cells are highly susceptible to diphtheria toxin-induced cytopathic effect (CPE) and can be used to efficiently determine the toxigenic status of clinical *Corynebacterium* isolates. (A) Vero cells were incubated with 2-fold serial dilutions of sterile-filtered bacterial supernatants obtained from C. ulcerans and *C. xerosis* cultures to determine the presence of diphtheria toxin as indicated by high CPE. (B) To confirm that the Vero cell CPE was due to diphtheria toxin and not due to an unrelated bacterial protein or other secreted factor, Vero cells were cultured in Medium alone (negative control), 10 CD_50_ of C. ulcerans supernatant (positive control), 10 CD_50_ of C. ulcerans supernatant incubated with a 1:10 dilution of naive serum from unvaccinated mice (DT-naive serum; i.e., a serum specificity control), or 10 CD_50_ of C. ulcerans supernatant incubated with a 1:10 dilution of immune serum from mice vaccinated against diphtheria toxoid (DT-immune serum) prior to addition to the Vero cell cultures. Complete neutralization of C. ulcerans supernatant-induced Vero cell cytotoxicity with DT-immune serum confirms that the agent causing Vero cell CPE is a form of diphtheria toxin. Bars represent the mean ± standard deviation from 2–8 replicates.

Based on the results of earlier diphtheria toxin-specific enzyme-linked immunosorbent assay (ELISA) assays, we had reason to believe that a toxigenic (i.e., diphtheria toxin-expressing) strain of *Corynebacterium* was circulating at the ONPRC. To investigate this in more detail, a group of 8 adult rhesus macaques (RM) that were born at ONPRC and pair-housed indoors while on an unrelated study were screened for Corynebacterium carriage at a single time point by swabbing the nares and streaking on differential media (Serum Tellurite agar plates). Small black colonies were streaked to isolation and speciated by matrix-assisted laser desorption/ionization time of flight mass spectrometry (MALDI-TOF), a technique that has 99.1% accuracy, 100% positive predictive value, and 100% negative predictive value for identification of toxigenic isolates of C. diphtheriae, C. pseudotuberculosis, and C. ulcerans (12). Using this approach, one animal was identified as an asymptomatic carrier of *C. xerosis*, a typically nontoxigenic strain of Corynebacterium that is rarely associated with overt human disease (13). Another clinically asymptomatic animal was confirmed by MALDI-TOF to have active carriage of C. ulcerans. To determine if either of these isolates expressed diphtheria toxin, bacterial cultures were grown at 37°C for 48 h and then the resulting bacterial supernatants were clarified and sterilized by 0.2 μM filtration prior to serial dilution and incubation with Vero cells, a cell line that is highly susceptible to diphtheria toxin-induced cytopathic effect (CPE) (Fig. 1) (14, 15) and represents the gold standard for determining toxigenic status (3). Culture supernatant from the *C. xerosis* strain showed no toxicity to Vero cells, indicating that it did not express the diphtheria toxin (Fig. 1A). In contrast, the C. ulcerans supernatant elicited high levels of CPE with a Cytotoxic Dilution-50% (CD_50_) of 2,636. This is within the range observed with other C. ulcerans isolates (CD_50_ = 512 to over 5,200) and C. diphtheriae isolates (CD_50_ = 80 to over 5,200) (15). To confirm that the Vero cell CPE was due specifically to diphtheria toxin, an antitoxin neutralization experiment was performed (Fig. 1B). Vero cells were incubated in medium alone as a negative control for determining background CPE or incubated with 10 CD50 of C. ulcerans supernatant as a positive control to demonstrate maximum CPE. If the C. ulcerans supernatant was pre-incubated with serum from unvaccinated/naive BALB/c mice (DT-naive), there was no reduction in Vero cell CPE. However, when the C. ulcerans supernatant was pre-incubated with neutralizing immune serum from BALB/c mice that had been vaccinated against diphtheria toxoid (DT-immune), then Vero cell CPE was prevented, and the cultures matched healthy Vero cell controls that were incubated in medium alone (Fig. 1B). Together, these neutralizing antibody studies confirm that the observed Vero cell CPE was specifically due to expression of a diphtheria toxin and not due to an unidentified bacterial protein or other secreted factor.

**FIG 2:**
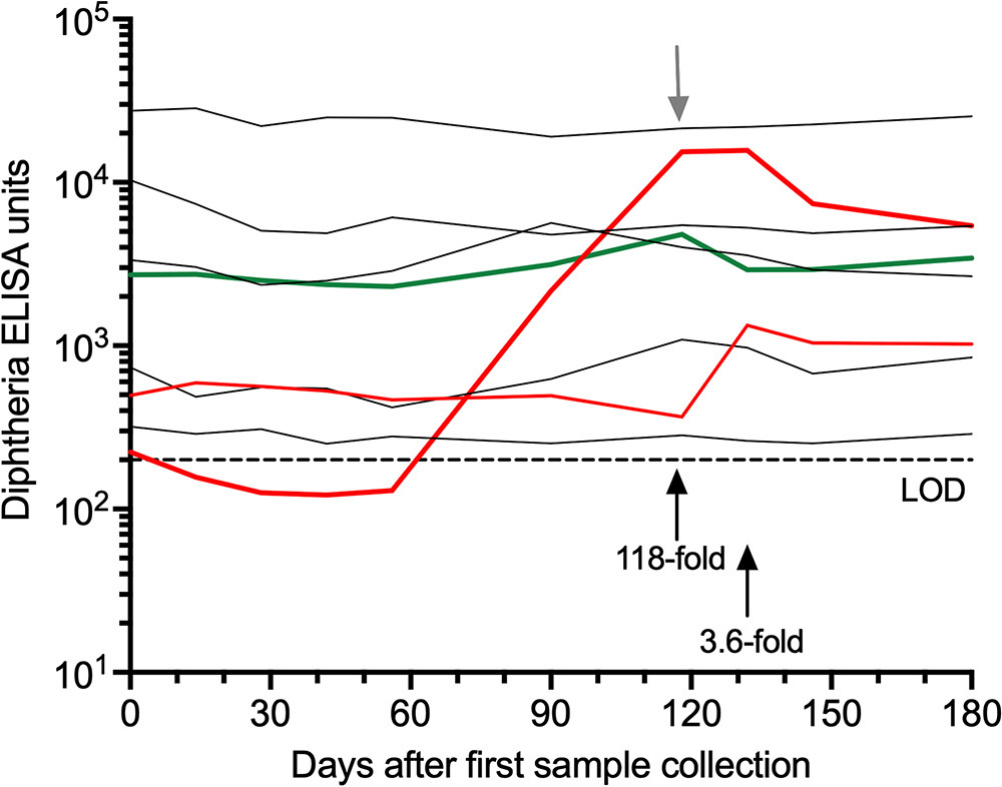
Evidence of toxigenic C. ulcerans transmission among indoor-housed rhesus macaques. A group of adult rhesus macaques (4.6–13.7 years of age) were housed indoors on an unrelated research project in which serum samples were being drawn every 2 to 4 weeks over a 6-month span of time. Diphtheria-specific antibody titers were measured by ELISA at the indicated time points. On day 120 after the first sample collection (downward gray arrow), nasal swabs were obtained for each animal and were plated on differential medium for Corynebacteria isolation (Serum Tellurite agar plates). A nontoxigenic strain of *C. xerosis* was isolated from one animal (green line) and C. ulcerans was isolated from an animal that had a 118-fold spike in anti-diphtheria antibodies (thick red line, upward black arrow). Another animal had a 3.6-fold serospike in anti-diphtheria antibodies (thin red line, upward black arrow) that occurred at approximately 2 weeks after the nasal swabs were obtained for analysis. LOD: Limit of detection.

Longitudinal analysis of diphtheria-specific serum antibodies from the 8 RM screened for Corynebacterium carriage provided further details regarding the timing of C. ulcerans infection (Fig. 2). First, seven of eight animals (88%) appeared to have been previously infected since they were seropositive for diphtheria-specific antibodies by ELISA at the beginning of the study. In contrast, one animal that was initially seronegative, as well as one of the animals with the lowest serum antibody levels at baseline, both showed antitoxin serospikes (118-fold and 3.6-fold increase in anti-diphtheria titers, respectively) indicative of a recent infection with subsequent antibody titers maintained at a higher plateau compared to the pre-spike serum samples obtained earlier. The previously seronegative animal with the highest serospike was also the one from which the C. ulcerans isolate was identified. Interestingly, these recently infected animals were housed in different buildings, indicating that C. ulcerans transmission was active in more than one location and was occurring in an indoor setting among pair-housed animals that are not socializing directly with one another in larger groups.

**FIG 3:**
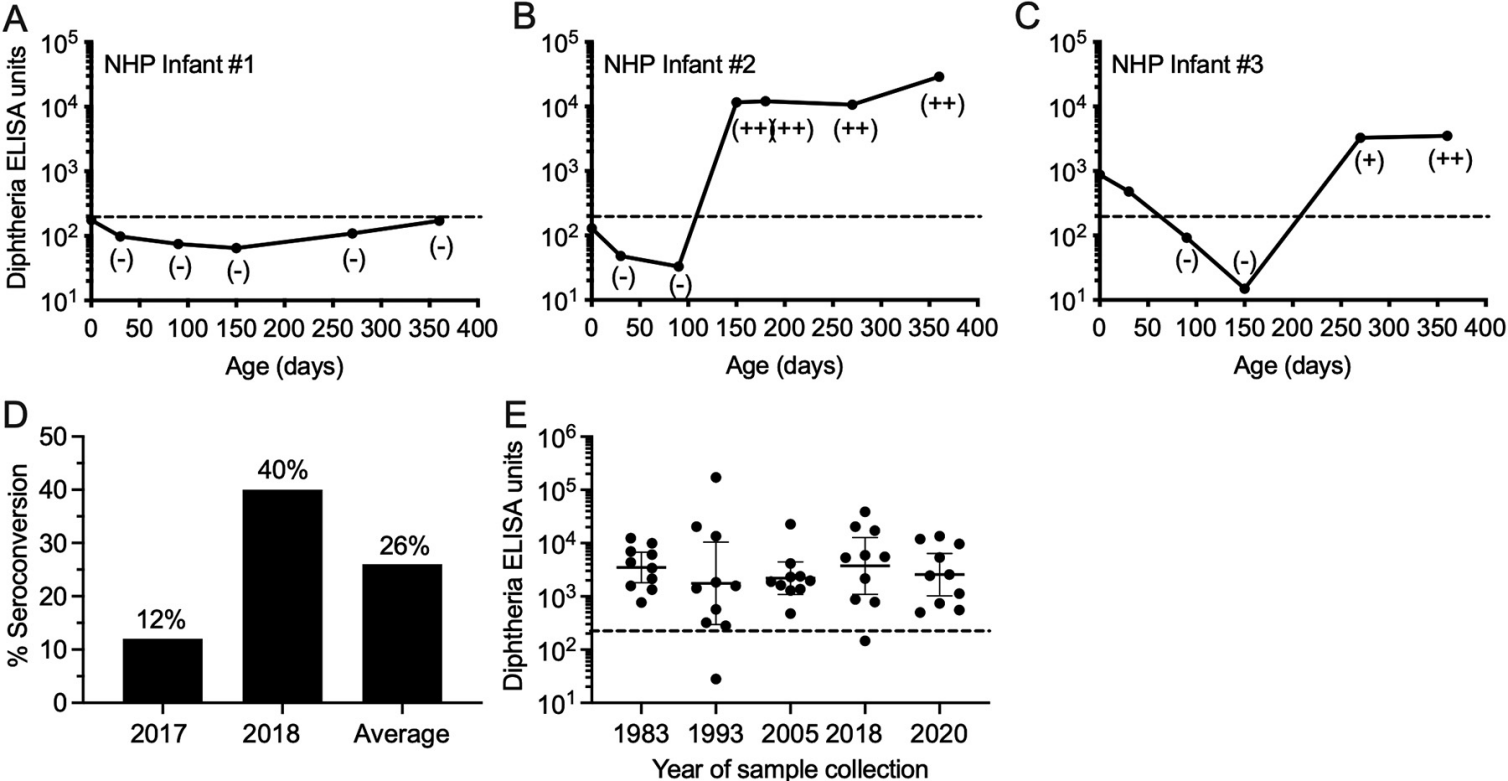
Diphtheria seroprevalence and annual seroconversion rates among rhesus macaques. Longitudinal analysis of diphtheria-specific antibody titers was performed among outdoor-housed infant rhesus macaques. The Day 0 antibody titers represent the serum antibody levels of the infant’s dam that were drawn when the infant was 30 days old and analysis of 109 infants revealed three different patterns of diphtheria-specific antibody responses (A–C). (A) NHP Infant #1 remained seronegative for the first year of observation. (B) NHP infant #2 was born to a seronegative dam but seroconverted by 5 months of age. (C) NHP Infant #3 was born to a diphtheria-seropositive dam and had maternal antibodies that waned to below the limits of detection (dashed line) by 3 months of age before the animal later seroconverted by 9 months of age. Samples were tested for diphtheria-specific neutralization activity and calibrated to the International Serum Standard (Diphtheria Antitoxin, NIBSC Code 00/496). Neutralizing antibody titers were noted by a negative sign (−) indicating <0.004 IU/mL (i.e., below the limit of detection), a positive sign (+) indicating ≥0.004 IU/mL, or a double positive sign (++) indicating ≥0.01 IU/mL. (D) Diphtheria-specific seroconversion rates were determined across two birth cohorts (representing 2017, 2018, or the average of both cohorts) among infants screened at 12 months of age. (E) Retrospective analysis of diphtheria-specific antibody responses among adult rhesus macaques were performed with 10 randomly chosen serum samples drawn during each of the indicated calendar years.

To determine the extent of annual toxigenic Corynebacterium transmission at the ONPRC, diphtheria toxin-specific antibody responses of outdoor-housed infant RM were analyzed by diphtheria toxin-specific ELISA and diphtheria toxin neutralization assays (Fig. 3). Analysis of longitudinal serum samples revealed different seroconversion patterns among the infant macaques. Some RM infants born to seronegative dams remained seronegative for up to a year of observation (Fig. 3A), others seroconverted shortly after birth (Fig. 3B), whereas other infants born to diphtheria-seropositive dams showed evidence of declining maternal antibodies followed by exposure events resulting in high and sustained antitoxin antibodies (Fig. 3C). Serum samples that scored positive by diphtheria toxin-specific ELISA also scored positive by diphtheria neutralizing assays, thereby confirming the specificity of the analysis and showing that these were biologically meaningful antibody titers above the protective threshold of ≥0.01 IU/mL. Active transmission resulted in 12% (6/51) of the 2017 RM birth cohort and 40% (23/58) of the 2018 RM birth cohort seroconverting within the first year after birth (average: 26% infant seroconversion by 1 year of age) (Fig. 3D). Bearing in mind that a sizeable proportion of infants were seroconverting each year, we examined banked serum samples from adult RM in 1983, 1993, 2005, and compared these to samples obtained in 2018 and 2020 (Fig. 3E). Remarkably, we found that diphtheria has been circulating among these NHP for decades with adult animals in each group demonstrating 90–100% seropositivity rates over the 37-year period of observation.

**FIG 4:**
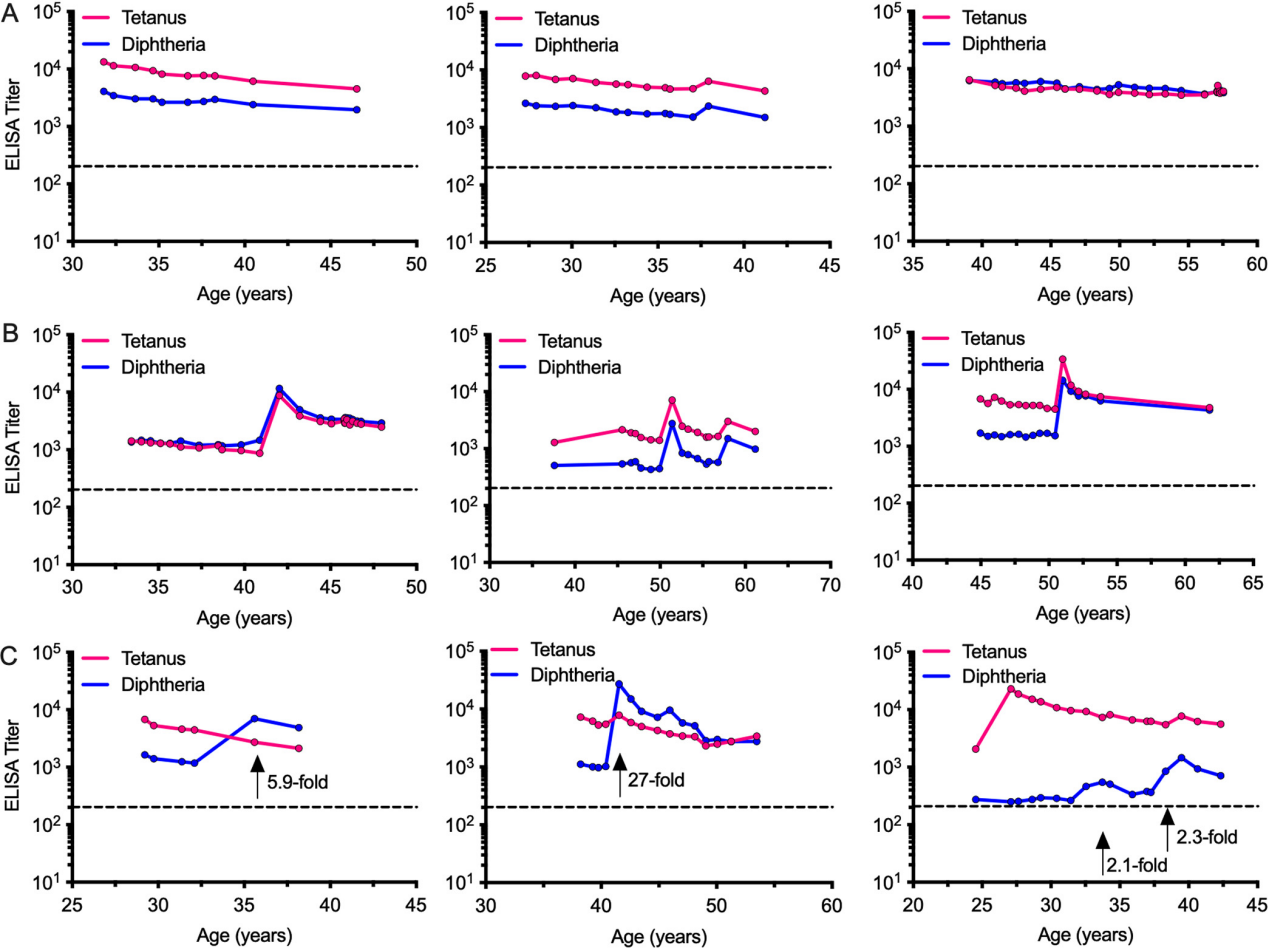
Representative antibody responses to tetanus and diphtheria among human subjects who have contact with NHP or NHP samples. In a previous study (16), serum samples from employees who had direct contact with NHP or NHP samples were analyzed for antibody responses to tetanus and diphtheria. These antibody responses followed three general patterns: (A) three examples of serum antibody responses to tetanus and diphtheria that declined slowly with similar kinetics in the absence of booster vaccination, (B) three examples of serum antibody responses to tetanus and diphtheria that show evidence of booster vaccination by serospikes to both vaccine antigens, (C) three examples of discordant serum antibody responses in which a spike in anti-diphtheria antibody responses is not accompanied by a concomitant serospike in anti-tetanus antibody responses, indicating a possible exposure to a toxigenic strain of Corynebacterium. Arrows indicate time points in which a serospike in diphtheria-specific antibody responses occurred and the numbers indicate the fold-increase in antibody titers. Dashed lines represent the limit of detection.

To determine if potential zoonotic transmission of toxigenic Corynebacterium to ONPRC personnel might have occurred, we reviewed longitudinal antibody responses from our prior seroepidemiology study (16). In this previous study, we found that in the absence of booster vaccination, antibodies to tetanus and diphtheria decay slowly with an 11-year to 19-year half-life, respectively (16) (Fig. 4A). Following booster vaccination, a spike in both tetanus- and diphtheria-specific antibodies is typically observed because although a tetanus-only vaccine was available in the past, tetanus and diphtheria toxoids are currently administered together in the same vaccine formulation (Fig. 4B). Identifying a serospike in diphtheria-specific antibodies in the absence of a parallel spike in tetanus-specific antibodies is therefore unusual because there has never been a commercially available diphtheria-only vaccine. Bearing this in mind, we identified 3 subjects who had antibody spikes that differed for tetanus and diphtheria (Fig. 4C). One individual had anti-diphtheria antibody responses spike by 5.9-fold in the absence of a concomitant spike in tetanus-specific antibodies that had instead declined by 39% over the same period of analysis (Fig. 4C, left panel). The serospike in ELISA titers to diphtheria were independently confirmed by diphtheria-specific neutralizing assays with titers increasing approximately 9-fold from 0.26 IU/mL to 2.37 IU/mL. Another subject (Fig. 4C, middle panel) showed an even larger 27-fold spike in anti-diphtheria antibodies that proceeded to decay more rapidly than that observed with the tetanus-specific antibodies. Prior studies have shown that antibody responses decline rapidly for the first 1–3 years after vaccination/exposure before regaining a more consistent long-term decay rate profile associated with that particular antigen (16, 17). In this case, following the anti-diphtheria spike, these antitoxin antibodies declined more rapidly over the next 3 years compared to the ant-tetanus antibodies measured at the same time points and this is in contrast to the post-spike antibody decay rates observed in Fig. 4B where the decay rate kinetics of anti-tetanus and anti-diphtheria antibodies mirrored each other. However, this 27-fold spike in anti-diphtheria antibodies was accompanied by a small 1.3-fold increase in anti-tetanus antibodies during the same time frame. Although this result differs sharply from the parallel tetanus/diphtheria serospikes and decay rates observed among the other participants presented in Fig. 4A and B, it is difficult to completely rule out a vaccination event and so we believe that this individual should be considered a suspect case of diphtheria rather than a probable case. A third subject (Fig. 4C, right panel) had an interesting serology profile because it appears that they may have been exposed to diphtheria on two different occasions. This person maintained low-level anti-diphtheria antibodies from 24 to 32 years of age before an increase in anti-diphtheria antibodies culminated in a total 2.1-fold peak over the initial antibody plateau. Note that at the time of the increase in anti-diphtheria titers, the anti-tetanus antibodies did not increase but instead continued a pattern of a modest 4% decay rate between these same time points. At the age of 38, this person had another 2.3-fold spike in anti-diphtheria antibodies that occurred in February of 1999, and again this serospike was not accompanied by an increase in anti-tetanus antibodies, since these antibodies declined by approximately 12% between these two time points. Later that year (October 1999) this person reported receiving a tetanus/diphtheria booster shot. This booster vaccination explains the further increase in anti-diphtheria antibodies that were observed in the graph along with a concomitant 1.4-fold rise in anti-tetanus antibodies that had not been observed during the earlier two diphtheria-only serospikes. Together, these results provide serological evidence indicating that there was at least one suspected case plus two more possible cases of individuals who had an exposure event involving a toxigenic strain of Corynebacteria.

## DISCUSSION

C. ulcerans is an emerging zoonotic agent of diphtheria, but the epidemiology of human C. ulcerans infections remains poorly understood. In these studies, we used serological assays to identify cases of diphtheria among captive-bred RM in addition to isolating a toxigenic strain of C. ulcerans from an infected adult macaque. Further serological studies indicated that toxigenic Corynebacteria have been circulating among these primates for at least 37 years and we identified up to three potential transmission events among humans who had longitudinal serum samples drawn due to their work involving direct contact with NHP or NHP samples.

In the pre-vaccine era from 1936 to 1945, there were approximately 21,000 cases of diphtheria and over 1,800 deaths per year in the United States. alone (18). Today, diphtheria is extremely rare in the United States. with <1 case reported per year in a country of over 300 million people (1, 11). Likewise, although large diphtheria epidemics in Europe resulted in as many as 1 million cases and 50,000 deaths in 1943 (19), today there are only a handful of cases or small clusters reported across Europe (4, 11). Although clinically severe diphtheria is generally characterized by the formation of an adherent pseudomembrane on the pharynx, tonsils, or larynx, less severe presentation of this respiratory disease may include pharyngitis, nasopharyngitis, tonsillitis, or laryngitis in the absence of pseudomembrane development or even some cases of asymptomatic carriage. Since diphtheria has become exceedingly rare in many industrialized countries, most clinicians are unlikely to have personally diagnosed a case and it is possible that without prompt identification, effective treatment may be delayed. For example, in a 1999 case study in the United States (1), a man presented to the emergency department with a 3-day history of sore throat, difficulty swallowing, and fever. He was prescribed erythromycin and sent home. He returned the next day with worsening difficulty swallowing and breathing and was hospitalized. Culture of throat specimens did not show typical C. diphtheriae morphology by methylene blue staining and diphtheria was excluded as a likely diagnosis. His condition rapidly deteriorated with development of pulmonary edema and despite intubation and mechanical ventilation, he died. Postmortem examination revealed a thick gray membrane that extended from the throat into the bronchial tree and a culture of toxigenic C. ulcerans was identified 4 days after the patient’s death. Another American case study from 2005 (1) began with a similar pattern of a woman complaining of fatigue, sore throat and difficulty swallowing. Her symptoms worsened and a throat examination showed an erythematous palate and edematous pharynx and uvula, with an exudate that extended into the nasopharynx. Following hospitalization and further worsening of respiratory symptoms, a tracheostomy was performed in which a thick, yellowish, fibrinous, sloughing membrane was noted to have coated the entire nasopharynx and extended into the trachea. The membrane was removed and with the suspicion of diphtheria, the patient was treated with 60,000 Units of diphtheria antitoxin (DAT). The patient made an uneventful recovery, and the throat swab specimens were later identified as C. ulcerans.

Rhesus macaques infected with C. ulcerans can present with severe respiratory diphtheria that mimics human disease including membranous involvement of the larynx and trachea, leading to mechanical asphyxia and death (7). In other rhesus macaque studies, respiratory diphtheria may not necessarily develop a recognizable pseudomembrane but C. ulcerans has been isolated from consolidated, necrotic lungs with focal abscesses, “containing yellowish, mucoid pus” (8). This description of a yellowish mucoid material in the infected macaque lung is not unlike the presentation of a lethal case of C. ulcerans in a human subject in 1988 in which the patient had “yellowish mucous covering the fauces and palate” and “At autopsy, the entire respiratory tree from the upper part of the larynx to the small bronchi was found to be covered by a thick yellowish membrane” (4). Although it is unclear what proportion of NHP respiratory disease may be attributable to C. ulcerans, the results described here indicate that this infection may be similar to certain clinical presentations of respiratory diphtheria in humans and represents a previously unappreciated model for studying naturally occurring Corynebacterium transmission.

This study has several limitations. Although a temporal relationship between a recent anti-diphtheria serospike and isolation of a toxigenic strain of C. ulcerans was established in rhesus macaques, investigation of human subjects was limited mainly to retrospective serological analysis of potential infection. Two of the human subjects in this study were lost to follow up and their NHP exposure history, travel history or exposure to farm animals or domestic pets could not be established. However, rates of active diphtheria transmission among NHP (Fig. 2 and 3) provides a plausible explanation for their anti-diphtheria toxin serospikes (Fig. 4). The third human subject with two anti-diphtheria serospikes (Fig. 4C, right panel) indicated that they had not traveled to a diphtheria-endemic country at the time of the potential exposures and had not been in direct contact with farm animals but owned a domestic house cat. Interestingly, this person did not have direct contact with NHP but worked extensively with NHP samples in a pathogen surveillance/diagnostic laboratory that performed routine serologies and viral and microbiological analysis of NHP specimens. This may be an important case in point because between 1987 and 2003, there were 3 separate incidents of laboratory-acquired C. diphtheriae infections among microbiologists documented in the United Kingdom (4), thus indicating a potential mechanism for contracting C. ulcerans infection in the absence of direct contact with infected NHP. Interestingly, this is not the first potential case of NHP-to-human transmission of an infectious agent at the ONPRC since there was serological evidence of transmission of a measles-like simian paramyxovirus to 4 ONPRC employees during an outbreak among the NHP in 1999 (16).

Although classic respiratory diphtheria has been characterized by a pseudomembrane that obstructs the airways, this is not always a clear diagnostic indicator of this rare disease. For example, previous studies have noted that none of 39 fully vaccinated diphtheria cases presented with a pseudomembrane, whereas 14 of 43 unvaccinated/incompletely vaccinated cases presented with classic respiratory diphtheria and development of a pseudomembrane (4). More recent studies also found that a pseudomembrane was only present among a quarter (one of four) of respiratory cases of toxigenic C. diphtheriae and only a quarter (two of eight) of respiratory cases linked to toxigenic C. ulcerans (2). Due to high vaccination coverage in the United States, those infected with diphtheria are less likely to present with severe symptoms of diphtheria and it is unclear how often asymptomatic or mildly symptomatic cases of infection without a pseudomembrane may go unreported. As noted previously (1), heightened clinical awareness of respiratory diphtheria-like illness caused by C. ulcerans is critical for early diagnosis and treatment and this study indicates that continued surveillance and use of appropriate safety precautions among individuals in close contact with NHP or NHP samples may be warranted.

## MATERIALS AND METHODS

### Subjects.

Serum samples were obtained from human subjects from the ONPRC as previously described (16). Samples were drawn on an approximately annual basis with extra blood samples drawn in the event of an exposure (e.g., bite or scratch) as part of a center-wide program to permit serological testing of individuals who worked closely with nonhuman primates or samples from nonhuman primates. The study was approved by the institutional review board of Oregon Health & Science University.

### Rhesus macaques.

The animal studies were performed in strict accordance with the recommendations described in the Guide for the Care and Use of Laboratory Animals of the National Institute of Health, the Office of Animal Welfare and the United States Department of Agriculture. All animal work was approved by the Oregon National Primate Research Center Institutional Animal Care and Use committee. The ONPRC has been continuously accredited by the American Association for Accreditation of Laboratory Animal Care since 1974 (PHS/OLAW Animal welfare Assurance #A3304-01).

### Isolation and identification of *Corynebacterium* species.

Nasal swab specimens were collected from rhesus macaques and cultured for microbial isolation on Serum Tellurite agar plates (BBL™ Serum Tellurite Agar L007411). The plates were incubated at 37°C for 48 h. A single colony was lifted from the primary isolation plate and streaked on to a Serum Tellurite Agar plate for further selective culture. A single colony was picked from the selection plate and streaked on to TSA-Agar for transport. Microbial identification was performed at EMSL Analytical, Inc. (EMSL code M256) by obtaining spectra using MALDI-TOF technology followed by analysis using the Vitek MS database.

### ELISA.

Quantitation of diphtheria and tetanus toxin-specific antibody levels was performed using a standardized enzyme-linked immunosorbent assay (ELISA) as previously described (16, 20) using optimized coating concentrations of tetanus toxoid (EMD Millipore) and diphtheria toxin (List Biological Laboratories).

### Diphtheria toxin-associated cytotoxicity testing of isolated *Corynebacterium* species.

Corynebacterium isolates were grown in BHI (Brain Heart Infusion) medium for 48 h at 37°C. The culture supernatants were filtered using 0.2 μM filter to remove bacteria and stored frozen at −80°C. Cytotoxic Dose-50 (CD_50_) for the Corynebacterium culture supernatant was determined using an *in vitro* Vero cell culture assay system (14, 15). The culture supernatant was serially 2-fold diluted in DMEM in 100 μL volume starting at 1:20 dilution in duplicate in flat-bottom tissue culture plates. Vero cells were added in 100 μL DMEM at a density of 2 × 10^4^ cells/well. The plate was then incubated at 37°C in a 6% CO_2_ incubator for 48 h. After the incubation, spent medium was removed, wells were gently washed with 200 μL PBS pH 7.4 to remove detached cells and cell debris. Cells were stained with 200 μL per well of 0.1% crystal violet solution in PBS containing 0.2% formaldehyde for 20 min. Excess dye was removed using soft running water. Plates were air-dried for 1 h. Bound stain was eluted using 200 μL of 70% ethanol (with 20 min of gentle shaking on a plate shaker) and transferred to an EIA grade polystyrene microtiter plate. Optical density (OD) at 595 nm was measured using an ELISA plate reader (Versa Max Plate Reader, Molecular Devices). A dose-response curve was generated by plotting the log-transformed values for diphtheria toxin concentration on *x* axis and OD in Y-axis. Serum dilution (NT_50_) corresponding to the average half-maximal OD was interpolated from the dilution curve by solving the linear equation y = mx + c.

### Diphtheria toxin neutralization assays.

Serum samples were serially 2-fold diluted starting at 1:10 dilution in 50 μL medium in a flat bottom microtiter tissue culture plate in duplicate. A challenge dose of 10 CD50 of culture supernatant (CS) of C. diphtheriae diphtheria toxin (List Biological Laboratories) was added to each well in a 50 μL volume, mixed by pipetting four times followed by incubation at 37°C in CO_2_ incubator for 2 h. Appropriate control wells were also set up including serum alone, no CS and CS alone. Following the incubation, 2 × 10^4^ Vero cells in 100 μL medium were added per well and the plate was further incubated at 37°C for 48 h. After the incubation, the plate was processed in the same method described in the CD_50_ determination protocol. After background subtraction, a dilution curve was generated by plotting the log-transformed values for serum dilution factor on *x* axis and OD on Y-axis. The average half-maximum OD was calculated from wells containing untreated Vero cells cultured in medium alone. Serum dilution neutralizing titer-50% (NT_50_) corresponding to the average half-maximum OD was interpolated from the linear portion of the dilution curve by solving the linear equation y = mx + c. Samples tested for neutralization activity were calibrated to the International Serum Standard (Diphtheria Antitoxin, NIBSC Code 00/496). Two naive serum samples from unvaccinated mice (NT_50_ < 10) and two DTaP (Tripedia, Aventis Pasteur, Inc.)-immune mouse serum samples were used in these studies. These mice were vaccinated intraperitoneally with 100 μL of Tripedia at day 0 and day 21 with serum samples collected at day 104 and had NT_50_ = 2,299 and 1,852, respectively.
